# Trends in Medical and Device Therapies Following Incident Heart Failure in Denmark during 1996–2019: A Nationwide Register-Based Follow-Up Study

**DOI:** 10.3390/jcdd10090362

**Published:** 2023-08-25

**Authors:** Asbjørn Ettrup-Christensen, Jawad H. Butt, Mikkel Porsborg Andersen, Maurizio Sessa, Christoffer Polcwiartek, Emil L. Fosbøl, Rasmus Rørth, Søren L. Kristensen, Christian Torp-Pedersen, Lars Køber, Morten Schou, Bhupendar Tayal, Peter Søgaard, Kristian Kragholm

**Affiliations:** 1 Department of Cardiology, Aalborg University Hospital, 9000 Aalborg, Denmark; a.ettrupchristensen@rn.dk (A.E.-C.); c.polcwiwiartek@rn.dk (C.P.); p.soegaard@rn.dk (P.S.); kdks@rn.dk (K.K.); 2 Department of Cardiology, Rigshospitalet, Copenhagen University Hospital, 2100 Copenhagen, Denmark; jawad_butt91@hotmail.com (J.H.B.); emil.fosboel@regionh.dk (E.L.F.); rasmusroerth@hotmail.com (R.R.); sorenlk@gmail.com (S.L.K.); lars.koeber.01@regionh.dk (L.K.); 3 Department of Cardiology, Nordsjaellands Hospital, 3400 Hillerød, Denmark; mikkel.porsborg.andersen@regionh.dk (M.P.A.); ctp@sund.ku.dk (C.T.-P.); 4 Department of Drug Design and Pharmacology, University of Copenhagen, 1172 Copenhagen, Denmark; 5 Department of Cardiology, Herlev-Gentofte Hospital, Copenhagen University Hospital, 2100 Copenhagen, Denmark; morten.schou.04@regionh.dk; 6 Department of Cardiology, Houston Methodist Hospital, Houston, TX 77030, USA; bhupendar.tayal@gmail.com; 7 Unit of Clinical Biostatistics and Epidemiology, Aalborg University Hospital, 9000 Aalborg, Denmark

**Keywords:** guideline-based, real-world patients, chronic heart failure

## Abstract

**Introduction:** Data on temporal trends in guideline-based medical and device therapies in real-world chronic heart failure (HF) patients are lacking. **Methods:** Register-based nationwide follow-ups of temporal trends in characteristics, guideline-recommended therapies, one-year all-cause mortality, and HF rehospitalizations in incident HF patients in Denmark during 1996–2019. **Results:** Among 291,720 incident HF patients, the age at the onset of HF was stable over time. While initially fairly equal, the sex distribution markedly changed over time with more incidents occurring in men overall. Hypertension and diabetes increased significantly over time, while other comorbidities remained stable. Between 1996 and 2019, significant increases in angiotensin-converting enzyme inhibitor and angiotensin II-receptor blocker (ACEi/ARB) therapy (38.2% to 69.9%), beta-blocker therapy (15.5% to 70.6%), and mineralocorticoid receptor antagonist (MRA) therapy (11.8% to 34.5%) were seen. Angiotensin receptor-neprilysin inhibitor (ARNI) and sodium-glucose cotransporter-2 inhibitors (SGLT2i) were introduced in the middle of the past decade, with minor increases but overall low uses: ARNI (2015: 0.1% vs. 2019: 3.9%) and SGLT2i (2012: <0.1% vs. 2019: 3.9%). Between 1999 and 2019, implantable cardioverter-defibrillator (ICD) use increased significantly: 0.1% to 3–4%. Cardiac resynchronization therapy (CRT) use similarly increased between 2000 and 2019: 0.2% to 2.3%. Between 1996 and 2019, one-year all-cause mortality decreased significantly: 34.6% to 20.9%, as did HF rehospitalizations (6% to 1.3%). **Conclusions:** Among 291,720 incident HF patients in Denmark during 1996–2019, significant increases in the use of ACEi/ARB, beta-blockers, MRAs, and devices were seen, with concurrent significant decreases in the one-year all-cause mortality and HF rehospitalization rates. The use of CRT, ARNI, and SGLT2i remained low, and MRAs were relatively underutilized, thereby representing future targets to potentially further improve HF prognoses.

## 1. Introduction

Approximately 64 million people suffer from heart failure (HF) worldwide [1]. HF is a major socioeconomic burden, and often leads to reduced quality of life, and increased morbidity and mortality. HF is one of the most frequent causes of hospitalization in patients over 65 years of age.

Over time, several landmark trials have been conducted, which represent cornerstones within guideline-recommended medical and device therapies in HF patients. These include angiotensin-converting enzyme (ACE) inhibitors/angiotensin II receptor antagonists (ARBs), beta-blocker therapy, and mineralocorticoid receptor antagonists (MRAs) as well as cardiac resynchronization therapy (CRT) and implantable cardioverter defibrillator (ICD) therapy [2,3,4,5]. In recent years, angiotensin receptor-neprilysin inhibitor (ARNI) and SGLT2 inhibitor (SGLT2i) drugs have emerged as potent, guideline-recommended medical therapies for patients with chronic heart failure, regardless of their diabetes status [6]. ACEis and ARBs showed beneficial effects in the CONSENSUS trial in 1987 [2], as did beta-blockers in 1999 in the CIBIS-II trial [3], MRAs in 1999 by Pitt et al. [4], CRT pacing by Leclercq et al. [5] in 1998, ARNIs by McMurray et al. [7] in 2014, and treatment with SGLT2i in 2019 by McMurray et al. [6]

In the management of HF, Schmidt et al. [8] showed that over the last three decades, there has been a reduction in morbidity and mortality, as well as fewer rehospitalizations. While that study focused on trends in patient characteristics, including comorbidities and clinical endpoints, there was no mention of the trends being used in guideline-based medication and device therapies over time. In general, there is a lack of studies focusing on trends in guideline-based medical and device therapies and outcomes in real-world chronic heart failure patients. As these therapies are important for the prognosis of patients, it is important to examine whether their uses have changed over time and whether there remains room for development to potentially improve patient outcomes further.

Therefore, the primary aim of our study was to describe trends in HF treatment in Denmark over three decades from 1996 to 2019. Our secondary aims were to describe trends in patient characteristics and outcomes including all-cause mortality and HF rehospitalization.

## 2. Methods

### 2.1. Population and Data Sources

We conducted a register-based nationwide follow-up study of patients with incident HF between 1996 and 2019 in Denmark. From the Danish Civil Registration System [9], we extracted unique personal registration numbers that are given to each Danish resident upon birth or immigration to link data between administrative registry sources, including the Danish National Patient Register [10] and the Danish National Prescription Registry [11].

We identified our source population based on the Danish Civil Registration System and included only adults ≥ 18 years of age. Through the Danish National Patient Register based on ICD-10 diagnosis codes used in hospitals in Denmark, we identified patients with HF. Thygesen et al. [12] showed that the use of ICD-10 has a positive predictive value of 100% for diagnosing HF. Patients were grouped according to whether they were diagnosed with HF during an inpatient contact (hospitalization) or whether it was an outpatient contact that led to the HF diagnosis. Both primary and secondary heart failure diagnosis codes were included. However, we only included heart failure diagnoses from cardiology departments or outpatient clinics. In addition, selected comorbidities were obtained using data from the Danish National Patient Registry and the Danish National Prescription Registry.

### 2.2. Study Variables

Hypertension, diabetes, and chronic pulmonary disease were identified by the Danish National Patient Registry and the Danish National Prescription Registry. Patients who were prescribed disease-specific drugs by either hospital-based physicians or general practitioners were all included in the disease-specific definitions; thus, we were able to identify patients as having one or more of these conditions without having been seen in hospitals, including hospital-based outpatient clinics for the specific condition. The following comorbidities were identified through the Danish National Patient Registry: myocardial infarction, ischemic heart disease, chronic kidney disease, stroke, and peripheral arterial disease.

### 2.3. Endpoints

Our primary endpoints were guideline-recommended medical and device therapies within 365 days of the HF diagnosis. Medical therapies included ACEi/ARB, beta-blockers, ivabradine, MRA, ARNI, and SGLT2i. In addition, device therapy included ICD and CRT. All of these medical therapies as well as device therapies, including ICD and CRT, were assessed within 365 days of the HF diagnosis.

Our secondary endpoints were one-year all-cause mortality and HF rehospitalizations. For patients who had HF diagnosed in an outpatient clinic, the first hospitalization due to HF was classified as rehospitalization due to HF.

### 2.4. Statistical Analysis

Continuous variables are shown using medians and first to third quartiles (Q1–Q3, 25–75th percentiles), and categorical variables using counts and percentages. Differences in continuous variables over time were tested using the Kruskal–Wallis test, while differences in categorical variables were compared using Pearson’s Chi-squared test. Cochrane-Armitage trend tests were performed to examine trends in the above-specified outcomes over time. A two-sided *p*-value < 0.05 was considered statistically significant. A sensitivity analysis among patients who were prescribed a combination of a beta-blocker and an ACEi/ARB was carried out, as a previous Danish study showed a higher accuracy of heart failure with systolic dysfunction among this group of patients [13]. Data management and analyses were performed using SAS version 9.4 (Cary, NC, USA) and R version 4.0.3 (Ref: R Core Team (2020). R: A language and environment for statistical computing. R Foundation for Statistical Computing, Vienna, Austria, https://www.R-project.org/ (accessed on 25 July 2023)).

## 3. Results

### 3.1. Patients

In total, 291,720 patients with an HF incident between 1996 and 2019 were included. Table 1 shows that between 10,287 and 14,655 patients each year were diagnosed with HF. Over time, an increasing number were diagnosed with HF in outpatient clinics compared to during hospitalization (19.3% in 1996 vs. 55.9% in 2018, and 50.3% in 2019).

### 3.2. Characteristics

The age of the patients was relatively stable over time, varying between 75 and 77 years. The sex distribution was fairly equal in the initial years, with 48% occurring in females and 52% in males, although over time, there was a marked change in this distribution, with 40% of patients being female and 60% male in 2019. The distribution of pre-existing hypertension and diabetes varied significantly over time, whereby 25% had hypertension in 1996 versus 66% in 2019, and 12% had diabetes in 1996 versus 21% in 2019. The distribution of other comorbidities was fairly constant over time (Table 1).

### 3.3. Temporal Trends in Guideline-Based HF Medical Therapies

**Figure 1:**
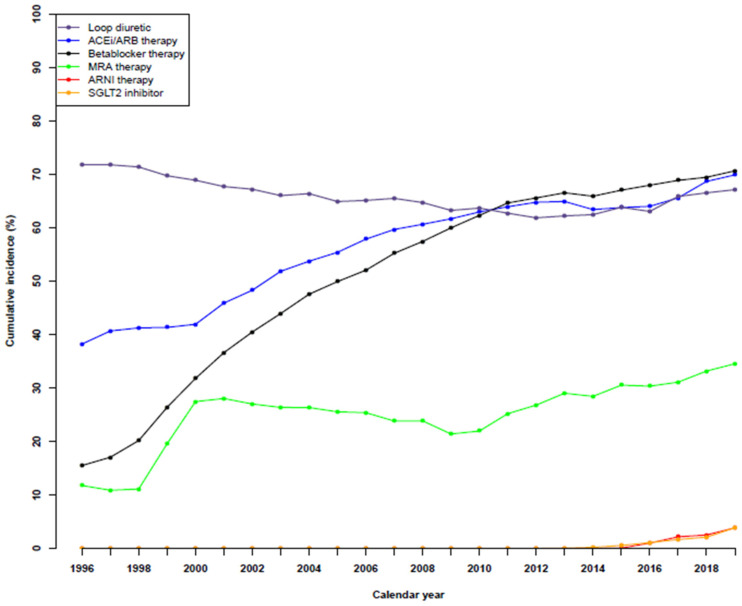
Temporal trends in guideline-based medical therapies in patients with heart failure during the period of 1996 to 2019.

Temporal trends in guideline-based HF medical therapies are shown in Figure 1 and for the specific use of drugs in Supplementary Material Figures S1–S4. In 1996, 71% were treated with loop diuretics. The number gradually fell to 61.8% in 2012 before rising slightly to 67.1% in 2019.

The number of patients receiving ACEi/ARB therapy increased significantly from 38.2% in 1996 to 69.9% in 2019. The same was observed for patients undergoing beta-blocker therapy, which increased from 15.5% to 70.6%, as well as for patients receiving MRA therapy, from 11.8% to 34.5%.

The first patients to be treated by ARNI therapy were in 2015 (0.1%), although the number of patients increased to 1.0%, 2.2%, 2.5%, and 3.9% in the following years. A similar trend was observed for patients receiving SGLT2i, whereby there was <0.1% in 2012, 0.1–0.2% in the two following years, 0.6% in 2015, 1.1% in 2016, 1.7% in 2017, 2.1% in 2018, and 3.9% in 2019. A sensitivity analysis of medical therapies up to two years after HF diagnosis showed a similar trend (Supplemental Figure S5).

### 3.4. Temporal Trends in ICD and CRT Device Use

**Figure 2:**
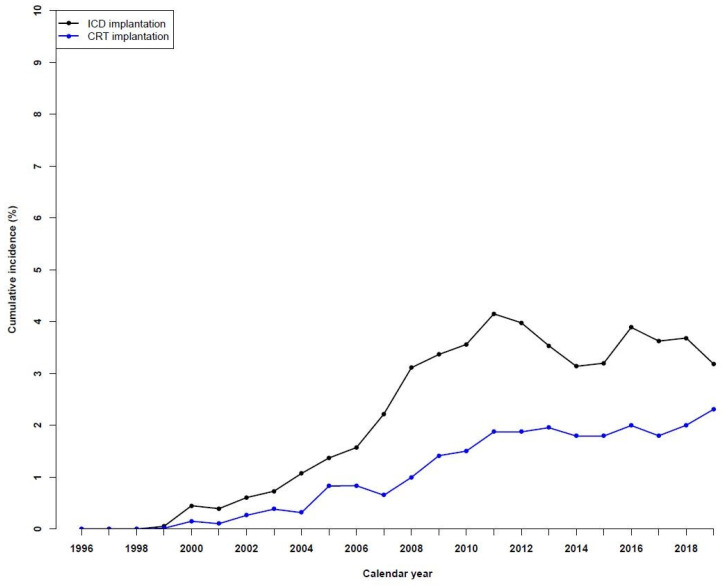
Temporal trends in device therapies in patients with heart failure during the period of 1996 to 2019.

In 1999, 0.1% of HF patients had an ICD implanted, while the number increased significantly to around 3–4% in the last years (Figure 2). A similar trend was observed for patients with a CRT implantation, with 0.2% in 2000, which increased to 2.3% in 2019. A sensitivity analysis of device therapies up to two years after HF diagnosis showed a similar trend (Supplemental Figure S6).

### 3.5. Temporal Trends in One-Year All-Cause Mortality and HF Rehospitalizations

**Figure 3:**
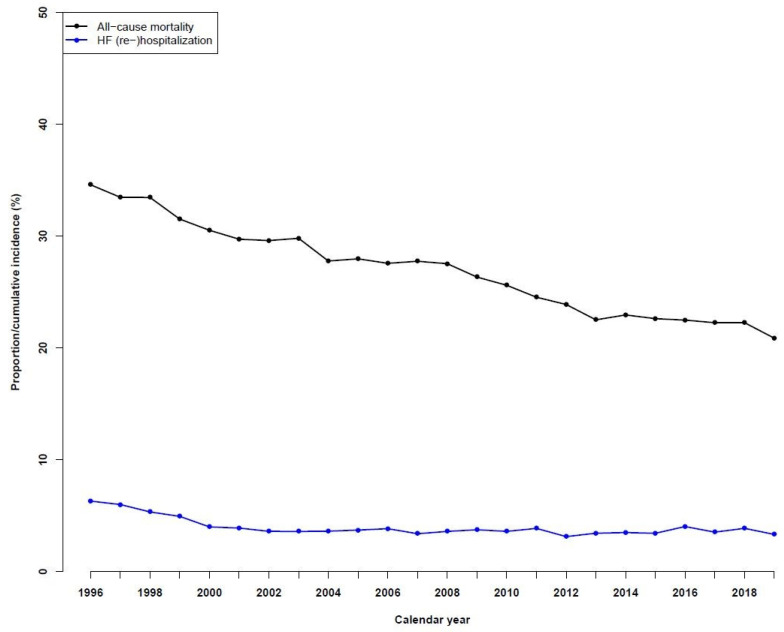
Temporal trends in all-cause mortality and heart failure (re)hospitalizations in patients with heart failure during the period of 1996 to 2019.

The one-year all-cause mortality decreased significantly from 1996 to 2019, decreasing from 34.6% in 1996 to 20.9% in 2019 (Figure 3). For one-year HF rehospitalizations, the incidences observed over time fell from 6.3% in 1996 to 3.3% in 2019.

### 3.6. Sensitivity Analysis in Patients Treated with a Beta-Blocker and an ACEi/ARB in Combination

The use of ARNI, SGLT2 inhibitors, ICD, and CRT was similar to the overall results of patients being treated with a beta-blocker and an ACEi or ARB in combination (Supplemental Figures S7 and S8).

## 4. Discussion

In this register-based nationwide follow-up study of patients with HF incidents in Denmark, including in patients from more than two decades ago (1996–2019), significant increases were observed in the use of ACEi/ARB therapy, beta-blocker therapy, and MRA therapy. ARNI and SGLT2i drugs were introduced in the middle of the previous decade and demonstrated minor increases in use but overall their use remained low at the end of the study period. In 1999, the first patients had an ICD implanted, and the number increased significantly from 0.1% to around 3–4% at the end of the study period. A similar trend was observed for patients with CRT implantations, although their overall use remained low at 2.3% in 2019 versus 0.2% in 2000. The one-year all-cause mortality fell significantly over the study period, from 34.6% in 1996 to 20.9% in 2019. The same trend was observed in the HF rehospitalization incidence during the study period, with 6.6% at the start versus 3.3% in 2019.

Similar rates of mortality, heart failure rehospitalization, and comorbidity distributions were observed in previous literature [8]. However, we also reported changes over time in the use of medical and device therapies and depicted these changes concurrently with changes in one-year mortality and HF rehospitalizations. The age of patients was stable over time, and the sex distribution was fairly equal initially, although, over the years, there was a marked change as the number of males increased. Pre-existing comorbidities, such as hypertension and diabetes, increased significantly over time, while other comorbidities remained fairly constant.

Concurrently with significant increases in the use of ACEi/ARB, beta-blockers, and MRAs over the past two decades, significant reductions in one-year mortality and HF rehospitalization rates were also observed. These changes were seen despite increasing comorbidity conditions, including diabetes and hypertension, and changes in the sex distribution of patients with HF, with more men than women being diagnosed with HF over time. The observed trends are especially important in the context of guidelines and evidence advocating for the use of decongestive drugs to improve patient prognoses [14]. In this context, a recent study focusing on MRA use alone, which was in agreement with our findings, indicates a steep increase in the use of MRAs, to just under 30% in all HF patients until around the year 2000, although, from that point onwards, only a further small increase was observed [15]. As this drug is contraindicated in patients with significant kidney disease, this may be a main reason for its lack of use, yet there is most likely a significant fraction of HF patients, without significant kidney disease, which could benefit from MRA treatment.

Although the use of ARNIs and SGLT2i has increased, the overall use remained relatively low, with both drugs being prescribed to 3.9% of HF patients in 2019. There are likely a number of reasons for the overall low use of these drugs, with one likely to be cost-related, as the cost of ARNIs is relatively expensive without drug reimbursement, while patients are not eligible for drug reimbursements until common HF medications, including ACEi/ARB, beta-blockers, and potassium-sparing drugs are fully up-titrated. In addition, specific criteria for the use of ARNIs, including NYHA-II to -IV, left ventricular ejection fraction ≤ 40%, and B-type natriuretic peptide levels are equivalent to those laid out in the PARADIGM-HF trial and have been applied in clinical practice, likely limiting the use of ARNIs [7]. However, these restrictions to the use of ARNIs have changed in recent guideline updates, and whether this has an impact on prognosis warrants further investigation when these data become available.

Several recent randomized controlled studies examining the effects of SGLT2 inhibitors have shown significant reductions in mortality and HF rehospitalizations [6]. In Denmark, it is only the most recent national guidelines for chronic heart failure treatment from earlier this year that have advocated for SGLT2 inhibitor drug use before patients were fully up-titrated with the common HF medications, including ACEi/ARB, beta-blockers, and MRA drugs. Previously, the cost has likely also been an issue, as reimbursement was not granted by the Danish Medicines Agency until early spring in 2021. Therefore, we expect that the use of SGLT2 inhibitors will have increased further in 2020 and 2021 and that the use will increase even further and more significantly in the future, given the change in guideline recommendations and drug reimbursement policy. Thus, it will be interesting to follow the future use of SGLT2i as well as trends in HF hospitalizations and mortality. Similarly, and lastly, despite significant increases in use, CRT also appears underutilized, as only 2.3% of HF patients were offered this treatment. The underutilization is substantial given approximately one-third of every HF patient has a left bundle branch block (LBBB) [16]. Therefore, in light of the evidence of CRT therapy in patients with HF and LBBB [17,18,19], we anticipate that HF prognosis may improve further following increases in the uses of ARNI, SGLT2i, and CRT in the coming years, although this warrants further investigation as these data become available.

## 5. Strengths and Limitations

The strength of this study includes its nationwide design, with a large real-world population included over many years and with negligible loss to follow-ups. Patients were unselected and representative of the entire population of Denmark. The Danish healthcare system is public and free of charge, and the use of private clinics is limited. As such, we believe our study provides a high internal validity that translates into high external validity, in particular settings with similar publicly financed healthcare systems in Europe. Nonetheless, the study has some limitations. The observational study design did not allow a causal link to be established between increases in guideline-based medical and device therapies and improved outcomes over time. Our registry sources lacked important clinical information, including echocardiography parameters and New York Heart Association (NYHA) symptom class data. However, despite the lack of echocardiographic data, it is important to stress that there is no tradition for coding heart failure with preserved left ventricular ejection fraction (HFpEF) as heart failure in the Danish National Patient Registry. Therefore, most patients with HF in our study can be assumed to have reduced left ventricular ejection fraction (LVEF) and our study findings should be interpreted in this context. HFpEF is a condition that can have one or multiple causes in interplay, including (but not exclusively): obesity, hypertension, diabetes mellitus, coronary artery disease, atrial fibrillation, chronic kidney disease, chronic obstructive pulmonary disease (COPD), and obstructive sleep apnea. Therefore, it is likely that the attending physician to a greater extent will code these disease entities without coding heart failure. In addition, most therapies are directed towards treating underlying conditions, with only a few drugs recommended for treating HFpEF, including loop diuretics and MRA, while the use of SGLT2 inhibitors has increased in recent years. Lastly, as echocardiography has been used to rule out reduced systolic ventricular function in these patients, it is not common practice to code heart failure in the presence of HFpEF in Denmark. A previous Danish study also underlined that HFpEF is underdiagnosed [13]. In addition, it stressed that the accuracy of heart failure with reduced ejection fraction (HFrEF) was higher in cases where a combination of a beta-blocker and an ACEi/ARB was prescribed. Therefore, we performed a sensitivity analysis among patients who were prescribed this combination and found that the use of MRAs as well as the use of SGLT2i and CRT were limited in these patients, which was in line with the overall results.

## 6. Conclusions

In this large nationwide study of 291,720 patients in Denmark with HF incidents during 1996–2019, significant increases in ACEi/ARB, beta-blocker, and MRA drug use and ICDs and CRTs were seen over time, concurrently with significant decreases in one-year all-cause mortality and HF rehospitalizations. Conversely, CRT, ARNI, and SGLT2i use remained low, while MRA use was relatively underutilized. However, increasing the use of these drugs as well as CRT may represent future tools to further improve HF prognoses.

## Figures and Tables

**Table 1:** Patient characteristics per calendar year in the study period.

	**1996**	**1997**	**1998**	**1999**	**2000**	**2001**	**2002**	**2003**
Variable	n = 12,209	n = 11,736	n = 12,555	n = 13,277	n = 14,655	n = 14,478	n = 14,344	n = 13,559
Age, median [Q1, Q3]	77 [69, 84]	77 [69, 84]	77 [69, 84]	77 [68, 84]	77 [68, 84]	77 [68, 84]	77 [68, 84]	77 [67, 84]
Male sex, n%	6284 (51.5)	6037 (51.4)	6456 (51.4)	6914 (52.1)	7593 (51.8)	7367 (50.9)	7413 (51.7)	7170 (52.9)
In-patient, n%	9847 (80.7)	9440 (80.4)	9893 (78.8)	10,030 (75.5)	10,888 (74.3)	10,474 (72.3)	10,136 (70.7)	9244 (68.2)
Hypertension, n%	3083 (25.3)	3486 (29.7)	4269 (34.0)	4857 (36.6)	5882 (40.1)	6302 (43.5)	6803 (47.4)	6808 (50.2)
Diabetes, n%	1528 (12.5)	1503 (12.8)	1660 (13.2)	1728 (13.0)	1972 (13.5)	1948 (13.5)	2087 (14.5)	1936 (14.3)
COPD, n%	1749 (14.3)	1752 (14.9)	1872 (14.9)	2160 (16.3)	2339 (16.0)	2444 (16.9)	2452 (17.1)	2267 (16.7)
Previous MI, n%	1955 (16.0)	1925 (16.4)	1999 (15.9)	2154 (16.2)	2429 (16.6)	2401 (16.6)	2583 (18.0)	2463 (18.2)
IHD, n%	3437 (28.2)	3374 (28.7)	3685 (29.4)	3937 (29.7)	4400 (30.0)	4433 (30.6)	4664 (32.5)	4518 (33.3)
Previous ICD, n%	0 (0.0)	0 (0.0)	0 (0.0)	0 (0.0)	9 (0.1)	22 (0.2)	21 (0.1)	52 (0.4)
CKD, n%	218 (1.8)	249 (2.1)	297 (2.4)	320 (2.4)	410 (2.8)	435 (3.0)	536 (3.7)	525 (3.9)
Stroke, n%	1395 (11.4)	1323 (11.3)	1459 (11.6)	1496 (11.3)	1666 (11.4)	1678 (11.6)	1685 (11.7)	1614 (11.9)
PAD, n%	1008 (8.3)	906 (7.7)	1052 (8.4)	1072 (8.1)	1273 (8.7)	1202 (8.3)	1278 (8.9)	1164 (8.6)
	**2004**	**2005**	**2006**	**2007**	**2008**	**2009**	**2010**	**2011**
Variable	n = 13,247	n = 12,544	n = 11,958	n = 11,803	n = 11,439	n = 11,166	n = 11,371	n = 11,154
Age, median [Q1, Q3]	77 [67, 84]	77 [66, 84]	76 [66, 84]	76 [65, 84]	76 [65, 84]	76 [66, 84]	76 [65, 84]	75 [65, 84]
Male sex, n%	7021 (53.0)	6777 (54.0)	6562 (54.9)	6496 (55.0)	6395 (55.9)	6258 (56.0)	6429 (56.5)	6403 (57.4)
In-patient, n%	8840 (66.7)	8129 (64.8)	7331 (61.3)	7251 (61.4)	6945 (60.7)	6376 (57.1)	6247 (54.9)	5938 (53.2)
Hypertension, n%	6955 (52.5)	7028 (56.0)	6833 (57.1)	6952 (58.9)	7066 (61.8)	7146 (64.0)	7254 (63.8)	7250 (65.0)
Diabetes, n%	2018 (15.2)	2034 (16.2)	1930 (16.1)	1948 (16.5)	2031 (17.8)	2052 (18.4)	2216 (19.5)	2179 (19.5)
COPD, n%	2275 (17.2)	2119 (16.9)	2084 (17.4)	2014 (17.1)	1977 (17.3)	1958 (17.5)	2043 (18.0)	2074 (18.6)
Previous MI, n%	2587 (19.5)	2455 (19.6)	2411 (20.2)	2378 (20.1)	2282 (19.9)	2262 (20.3)	2434 (21.4)	2372 (21.3)
IHD, n%	4697 (35.5)	4439 (35.4)	4336 (36.3)	4212 (35.7)	4112 (35.9)	4113 (36.8)	4291 (37.7)	4230 (37.9)
Previous ICD, n%	53 (0.4)	61 (0.5)	66 (0.6)	101 (0.9)	115 (1.0)	96 (0.9)	129 (1.1)	112 (1.0)
CKD, n%	546 (4.1)	558 (4.4)	564 (4.7)	591 (5.0)	625 (5.5)	669 (6.0)	685 (6.0)	708 (6.3)
Stroke, n%	1621 (12.2)	1583 (12.6)	1490 (12.5)	1491 (12.6)	1474 (12.9)	1434 (12.8)	1501 (13.2)	1431 (12.8)
PAD, n%	1225 (9.2)	1214 (9.7)	1134 (9.5)	1083 (9.2)	1059 (9.3)	1106 (9.9)	1161 (10.2)	1180 (10.6)
	**2012**	**2013**	**2014**	**2015**	**2016**	**2017**	**2018**	**2019**
Variable	n = 11,310	n = 11,507	n = 11,370	n = 11,816	n = 11,515	n = 11,169	n = 10,287	n = 11,251
Age, median [Q1, Q3]	75 [65, 84]	75 [65, 83]	75 [66, 83]	75 [66, 84]	75 [66, 83]	75 [66, 84]	75 [66, 83]	75 [65, 83]
Male sex, n%	6612 (58.5)	6780 (58.9)	6647 (58.5)	6930 (58.6)	6745 (58.6)	6587 (59.0)	6236 (60.6)	6725 (59.8)
In-patient, n%	5643 (49.9)	5495 (47.8)	5409 (47.6)	5570 (47.1)	5487 (47.7)	5032 (45.1)	4541 (44.1)	5589 (49.7)
Hypertension, n%	7445 (65.8)	7640 (66.4)	7630 (67.1)	7855 (66.5)	7665 (66.6)	7525 (67.4)	6715 (65.3)	7462 (66.3)
Diabetes, n%	2296 (20.3)	2387 (20.7)	2309 (20.3)	2484 (21.0)	2410 (20.9)	2368 (21.2)	2082 (20.2)	2370 (21.1)
COPD, n%	1988 (17.6)	2046 (17.8)	2121 (18.7)	2120 (17.9)	2151 (18.7)	2103 (18.8)	1909 (18.6)	2029 (18.0)
Previous MI, n%	2380 (21.0)	2256 (19.6)	2237 (19.7)	2293 (19.4)	2144 (18.6)	1977 (17.7)	1807 (17.6)	1808 (16.1)
IHD, n%	4249 (37.6)	4143 (36.0)	4065 (35.8)	4125 (34.9)	3892 (33.8)	3623 (32.4)	3213 (31.2)	3272 (29.1)
Previous ICD, n%	154 (1.4)	144 (1.3)	161 (1.4)	156 (1.3)	157 (1.4)	155 (1.4)	134 (1.3)	212 (1.9)
CKD, n%	818 (7.2)	828 (7.2)	881 (7.7)	941 (8.0)	908 (7.9)	915 (8.2)	863 (8.4)	859 (7.6)
Stroke, n%	1522 (13.5)	1391 (12.1)	1375 (12.1)	1496 (12.7)	1438 (12.5)	1349 (12.1)	1167 (11.3)	1182 (10.5)
PAD, n%	1132 (10.0)	1204 (10.5)	1193 (10.5)	1258 (10.6)	1181 (10.3)	1137 (10.2)	1007 (9.8)	1016 (9.0)

Abbreviations: n: number; Q1, Q3: 1st, 3rd quartiles; COPD: chronic obstructive pulmonary disease; MI: myocardial infarction; IHD: ischemic heart disease; ICD: implantable cardioverter defibrillator; CKD: chronic kidney disease; PAD: peripheral artery disease.

## Data Availability

Data are stored on secure servers on Statistics Denmark and cannot be shared according to Statistics Denmark reg-ulations. Access to Statistics Denmark servers and the associated data can be granted by Statistics Denmark upon adequate permissions.

## Supplementary Materials

The following supporting information can be downloaded at: https://www.mdpi.com/article/10.3390/jcdd10090362/s1, Figure S1: Temporal trends in guideline-based heart failure medical therapies for diuretics during the period 1996 to 2019; Figure S2: Temporal trends in guideline-based heart failure medical therapies for angiotensin converting enzyme inhibitors during the period 1996 to 2019; Figure S3: Temporal trends in guideline-based heart failure medical therapies for angiotensin II receptor antagonists during the period 1996 to 2019; Figure S4: Temporal trends in guideline-based heart failure medical therapies for beta-blocker or ivabradine therapy during the period 1996 to 2019; Figure S5: Temporal trends in guideline-based medical therapies up to two years after heart failure diagnosis during the period 1996 to 2019; Figure S6: Temporal trends in device therapies up to two years after heart failure diagnosis during the period 1996 to 2019; Figure S7: Temporal trends in guideline-based heart failure medical therapies among patients treated with a combination of a beta-blocker and an ACEi/ARB during the period 1996 to 2019; Figure S8: Temporal trends in device therapies among patients treated with a combination of a beta-blocker and an ACEi/ARB during the period 1996 to 2019.
