# Supplementary material for: Trends in Medical and Device Therapies Following Incident Heart Failure in Denmark during 1996–2019: A Nationwide Register-Based Follow-Up Study
Source: J Cardiovasc Dev Dis. 2023 Aug 25;10(9):362. doi: 10.3390/jcdd10090362 (PMC10531766; doi:10.3390/jcdd10090362)

Supplementary Material

Trends in Medical and Device Therapies Following Incident Heart Failure in Denmark during 1996–2019: A Nationwide Register-Based Follow-Up Study

Asbjørn Ettrup-Christensen [1], Jawad H. Butt [2], Mikkel Porsborg Andersen [3], Maurizio Sessa [4,*], Christoffer Polcwiartek [1], Emil L. Fosbøl [2], Rasmus Rørth [2], Søren L. Kristensen [2], Christian Torp-Pedersen [3], Lars Køber [2], Morten Schou [5], Bhupendar Tayal [6], Peter Søgaard [1] and Kristian Kragholm [1,7]

[1] Department of Cardiology, Aalborg University Hospital, 9000 Aalborg, Denmark; a.ettrupchristensen@rn.dk (A.E.-C.); c.polcwiwiartek@rn.dk (C.P.); p.soegaard@rn.dk (P.S.); kdks@rn.dk (K.K.)
[2] Department of Cardiology, Rigshospitalet, Copenhagen University Hospital, 2100 Copenhagen, Denmark; jawad_butt91@hotmail.com (J.H.B.); emil.fosboel@regionh.dk (E.L.F.); rasmusroerth@hotmail.com (R.R.); sorenlk@gmail.com (S.L.K.); lars.koeber.01@regionh.dk (L.K.)
[3] Department of Cardiology, Nordsjaellands Hospital, 3400 Hillerød, Denmark; mikkel.porsborg.andersen@regionh.dk (M.P.A.); ctp@sund.ku.dk (C.T.-P.)
[4] Department of Drug Design and Pharmacology, University of Copenhagen, 1172 Copenhagen, Denmark
[5] Department of Cardiology, Herlev-Gentofte Hospital, Copenhagen University Hospital, 2100 Copenhagen, Denmark; morten.schou.04@regionh.dk
[6] Department of Cardiology, Houston Methodist Hospital, Houston, TX 77030, USA; bhupendar.tayal@gmail.com
[7] Unit of Clinical Biostatistics and Epidemiology, Aalborg University Hospital, 9000 Aalborg, Denmark
* Correspondence: maurizio.sessa@sund.ku.dk

Supplementary Figure S1

Temporal trends in guideline-based heart failure medical therapies for diuretics during the period 1996 to 2019.

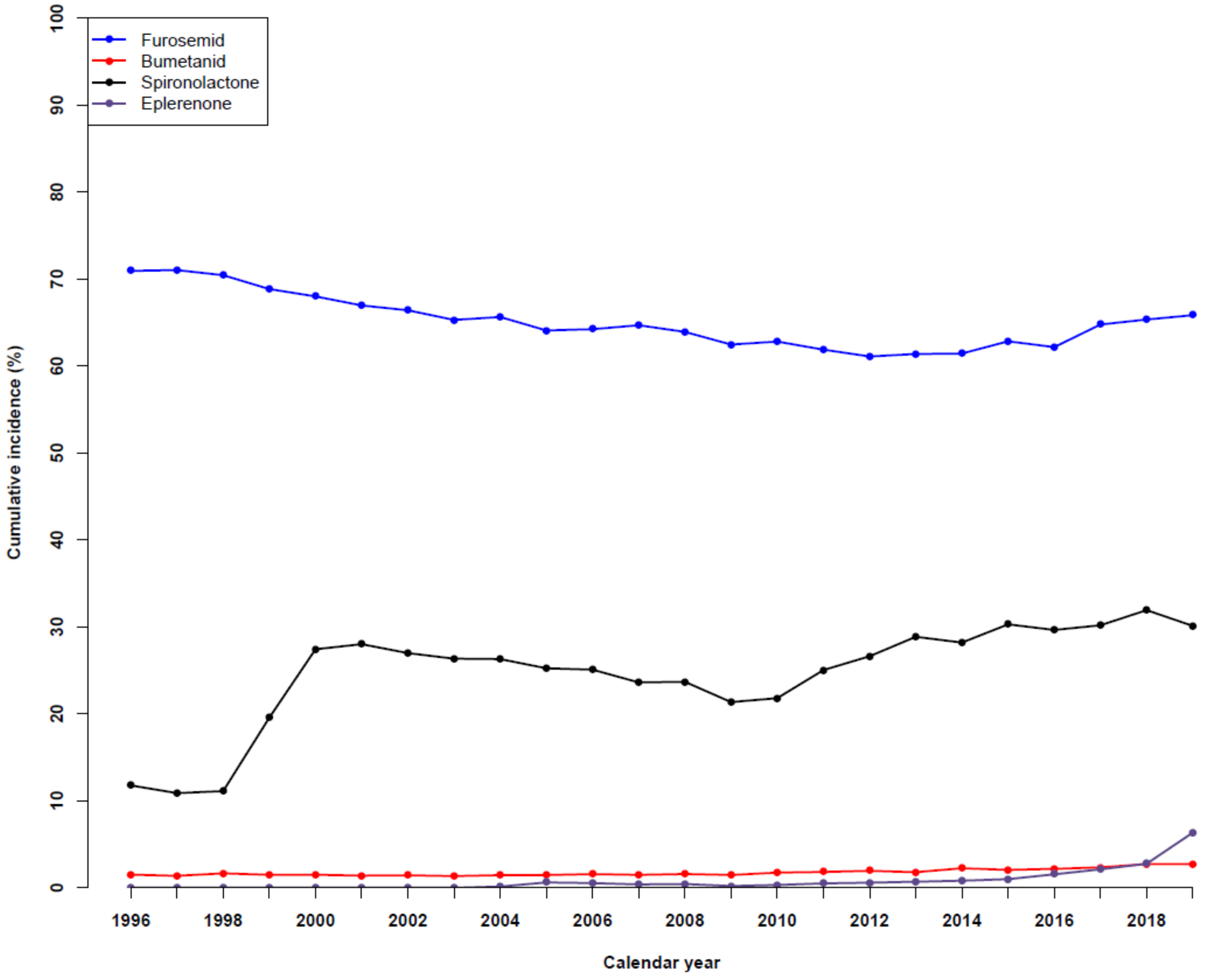

Supplementary Figure S2

Temporal trends in guideline-based heart failure medical therapies for angiotensin converting enzyme inhibitors during the period 1996 to 2019.

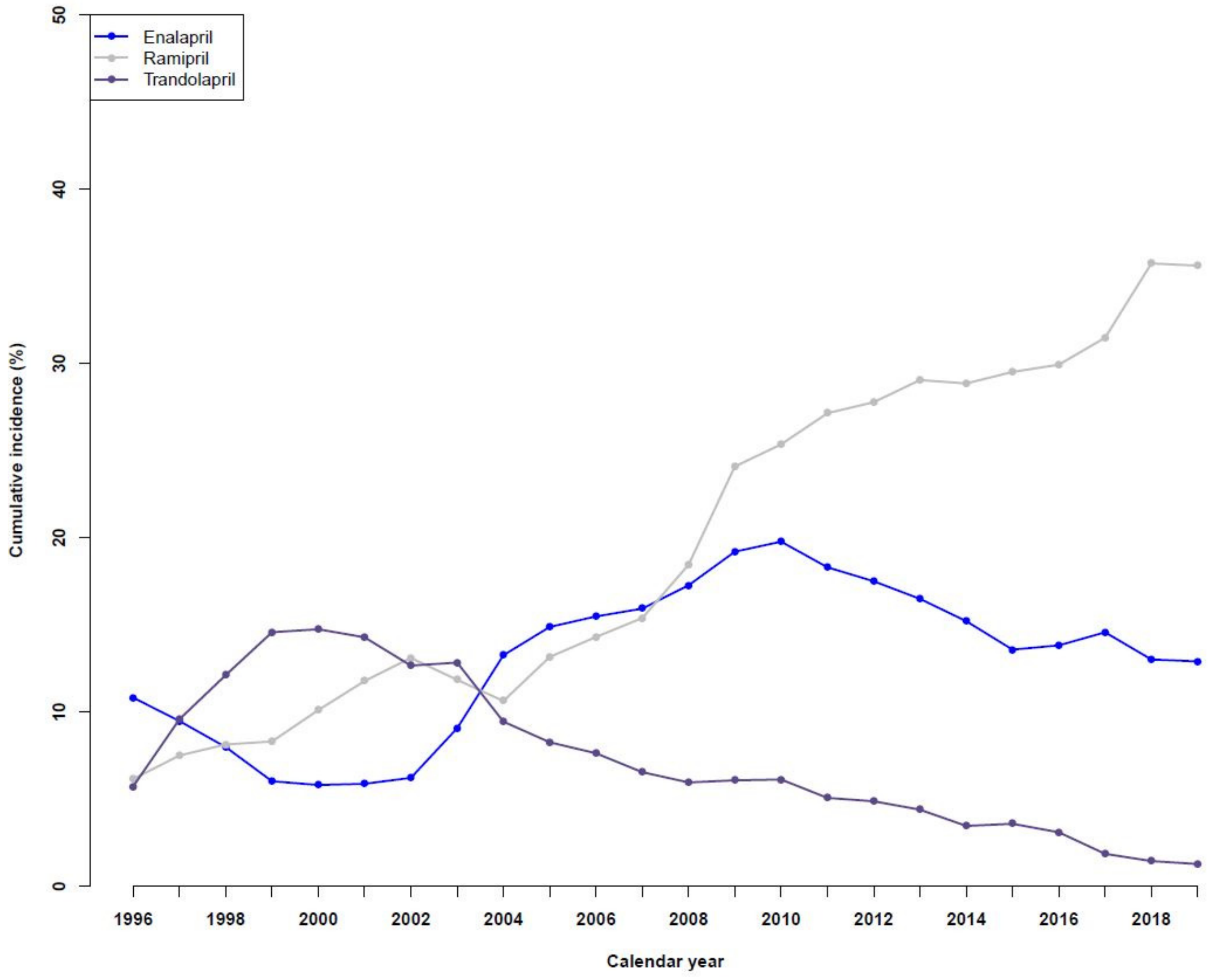

Supplementary Figure S3

Temporal trends in guideline-based heart failure medical therapies for angiotensin II receptor antagonists during the period 1996 to 2019.

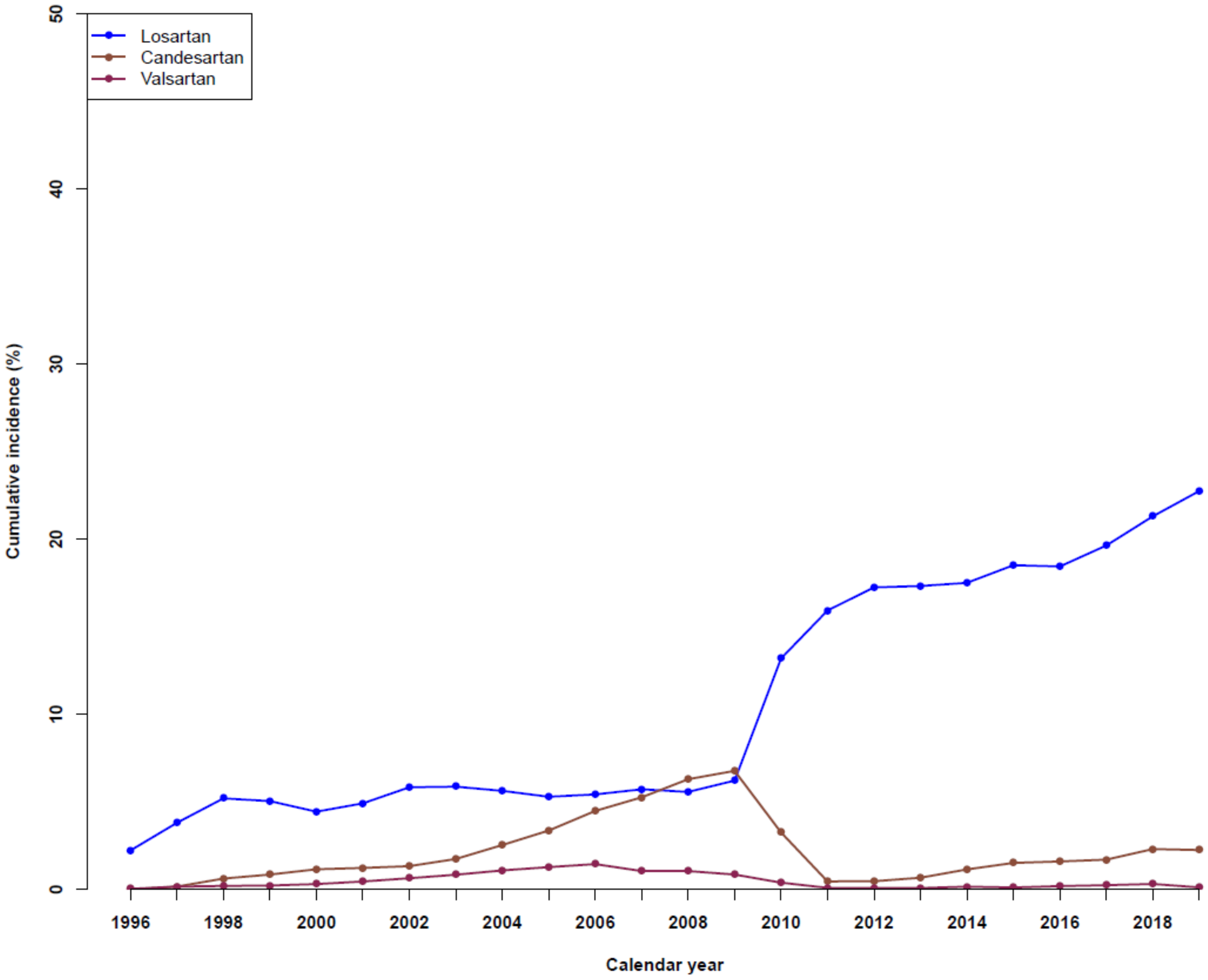

Supplementary Figure S4

Temporal trends in guideline-based heart failure medical therapies for beta-blocker or ivabradine therapy during the period 1996 to 2019.

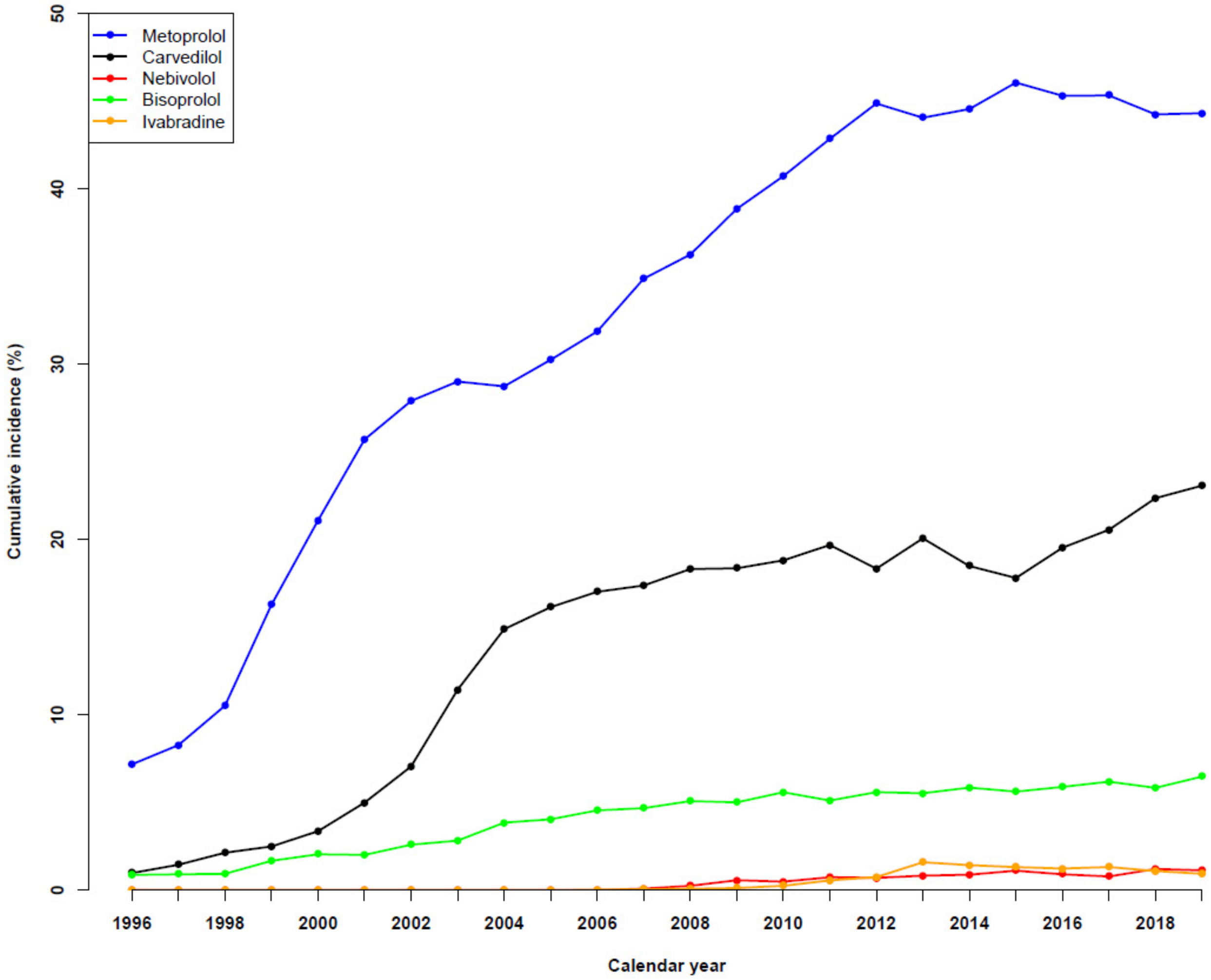

Supplementary Figure S5

Temporal trends in guideline-based medical therapies up to two years after heart failure diagnosis during the period 1996 to 2019.

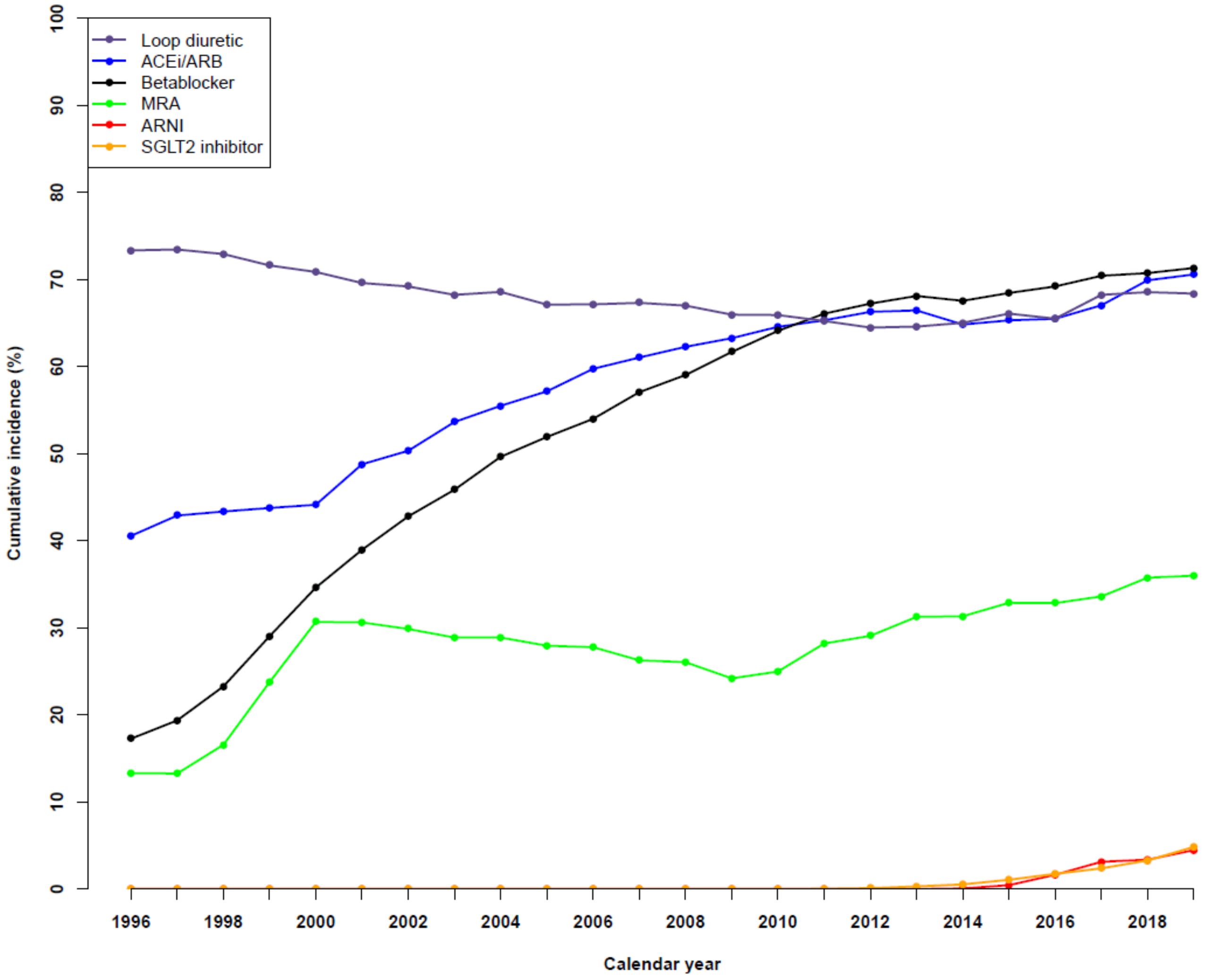

Supplemental Figure S6

Temporal trends in device therapies up to two years after heart failure diagnosis during the period 1996 to 2019.

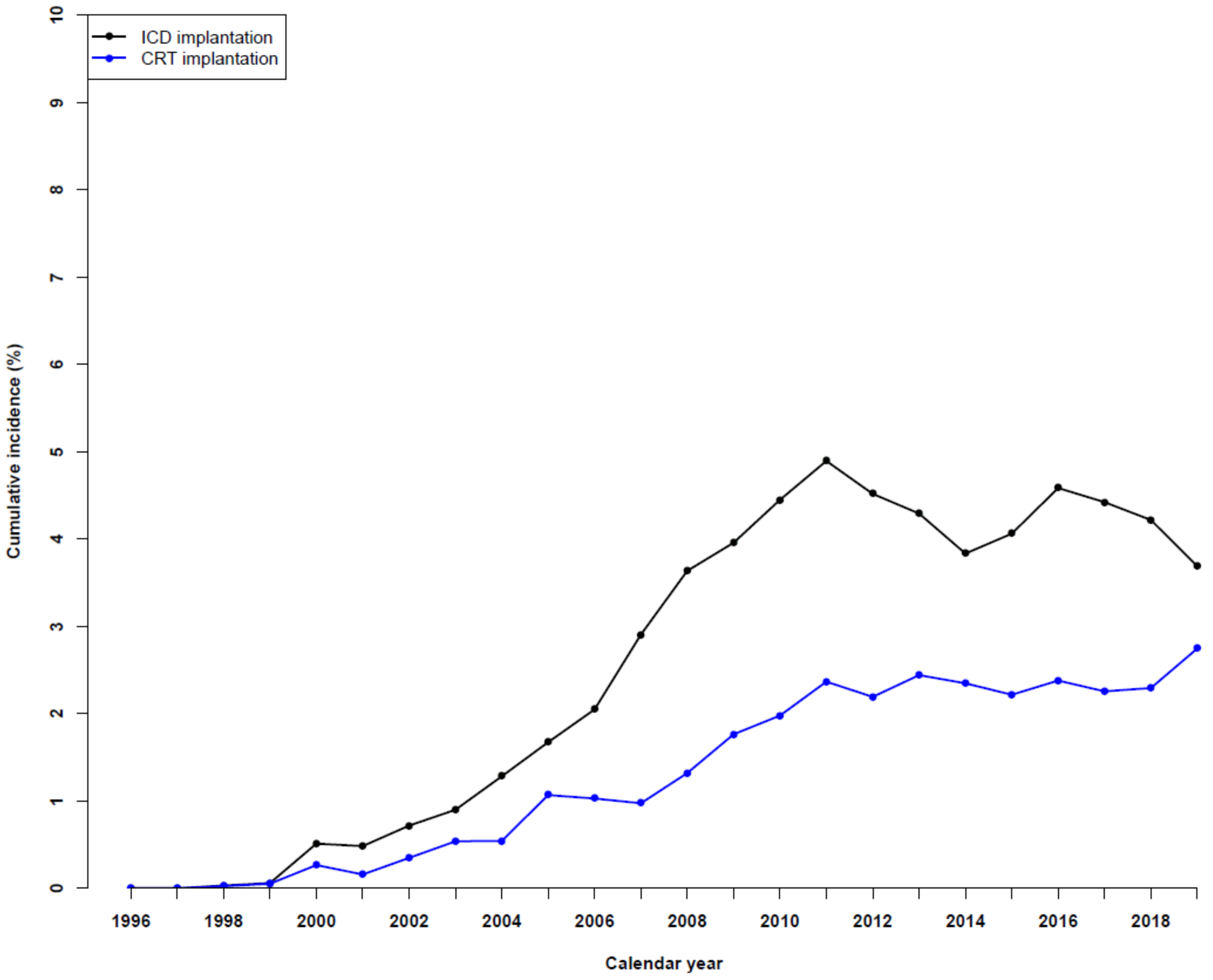

Supplementary Figure S7

Temporal trends in guideline-based heart failure medical therapies among patients treated with a combination of a beta-blocker and an ACEi/ARB during the period 1996 to 2019.

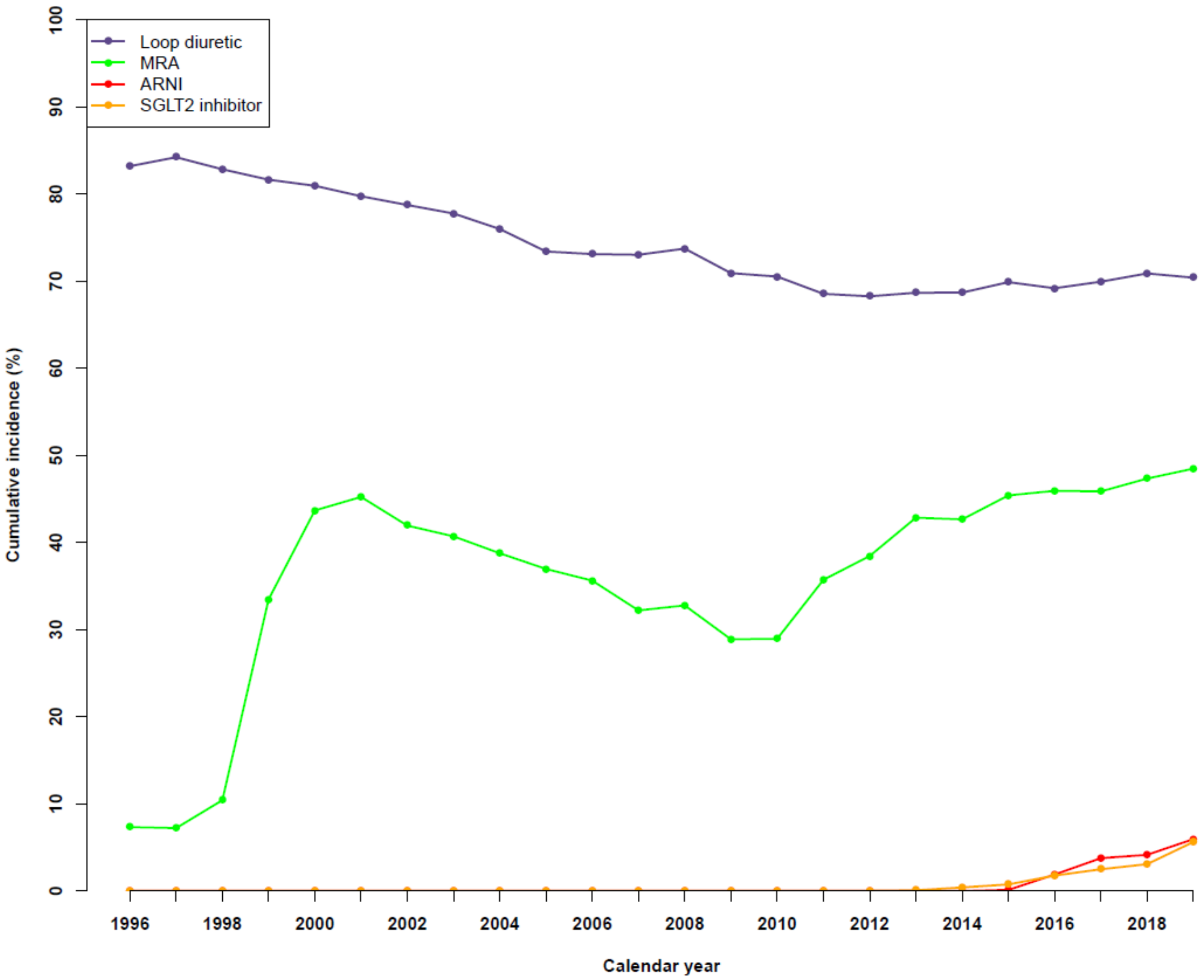

Supplementary Figure S8

Temporal trends in device therapies among patients treated with a combination of a beta-blocker and an ACEi/ARB during the period 1996 to 2019.

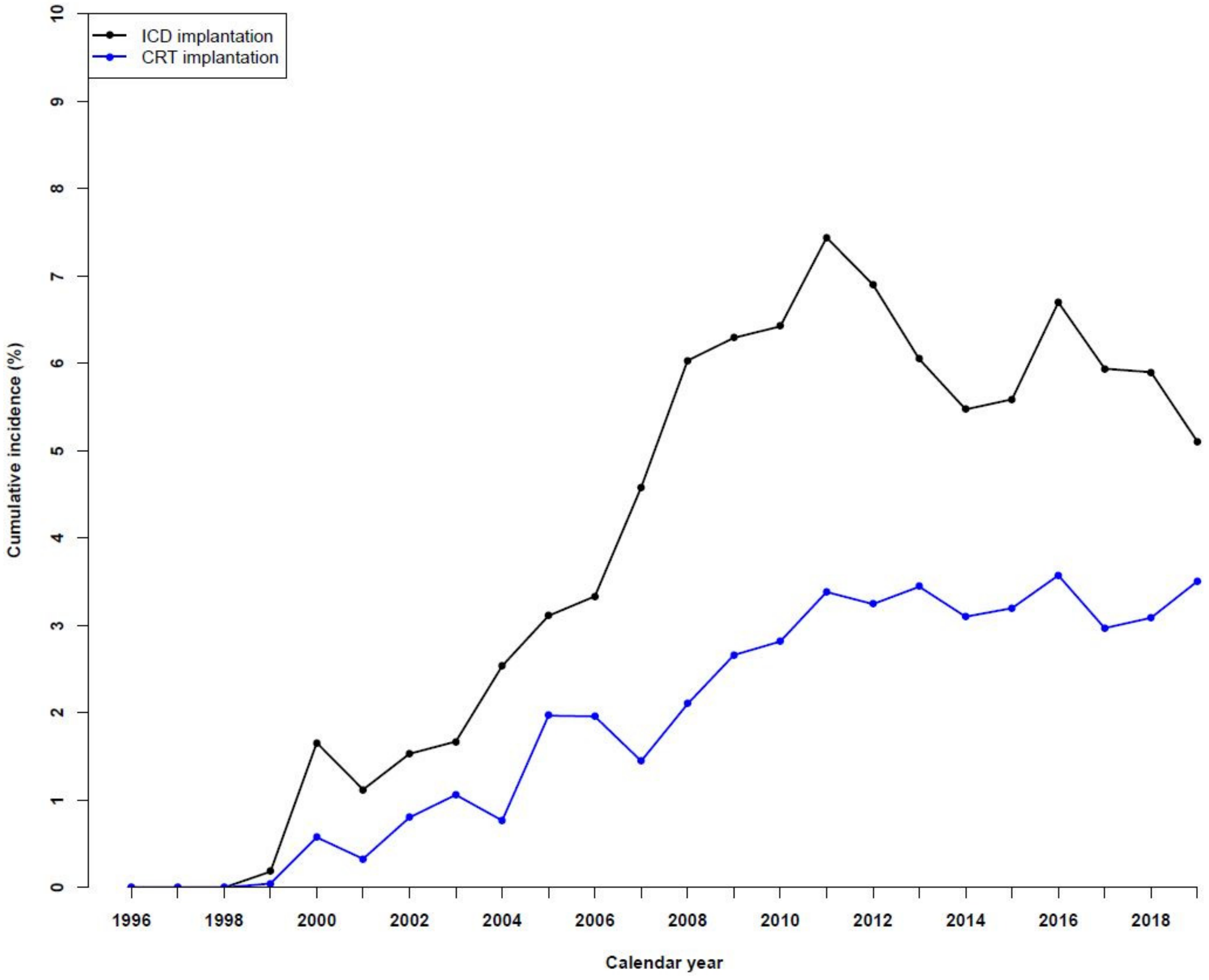

Supplement: Supplementary file 1 [file jcdd-10-00362-s001.zip › jcdd-2552860-supplementary.pdf]
